# Effects of Study Design and Allocation on participant behaviour - ESDA: study protocol for a randomized controlled trial

**DOI:** 10.1186/1745-6215-12-42

**Published:** 2011-02-14

**Authors:** Kypros Kypri, Jim McCambridge, Amanda Wilson, John Attia, Paschal Sheeran, Steve Bowe, Tina Vater

**Affiliations:** 1 Centre for Clinical Epidemiology & Biostatistics, School of Medicine and Public Health, University of Newcastle, Callaghan NSW 2308, Australia; 2 London School of Hygiene & Tropical Medicine, London, UK; 3 Department of Psychology, University of Sheffield, UK; 4 Cancer Behaviour Research Centre, Victoria, Australia; 5 Injury Prevention Research Unit, Department of Preventive & Social Medicine, University of Otago, New Zealand

## Abstract

**Background:**

What study participants think about the nature of a study has been hypothesised to affect subsequent behaviour and to potentially bias study findings. In this trial we examine the impact of awareness of study design and allocation on participant drinking behaviour.

**Methods/Design:**

A three-arm parallel group randomised controlled trial design will be used. All recruitment, screening, randomisation, and follow-up will be conducted on-line among university students. Participants who indicate a hazardous level of alcohol consumption will be randomly assigned to one of three groups. Group A will be informed their drinking will be assessed at baseline and again in one month (as in a cohort study design). Group B will be told the study is an intervention trial and they are in the control group. Group C will be told the study is an intervention trial and they are in the intervention group. All will receive exactly the same brief educational material to read. After one month, alcohol intake for the past 4 weeks will be assessed.

**Discussion:**

The experimental manipulations address subtle and previously unexplored ways in which participant behaviour may be unwittingly influenced by standard practice in trials. Given the necessity of relying on self-reported outcome, it will not be possible to distinguish true behaviour change from reporting artefact. This does not matter in the present study, as any effects of awareness of study design or allocation involve bias that is not well understood. There has been little research on awareness effects, and our outcomes will provide an indication of the possible value of further studies of this type and inform hypothesis generation.

**Trial Registration:**

Australia and New Zealand Clinical Trials Register (ANZCTR): ACTRN12610000846022

## Background

The "Hawthorne effect", also known as *reactivity*, refers to the possibility that people may change their behaviour simply because they know they are participating in a study [1]. The name derives from studies done in the workplace at the Western Electric Plant in Hawthorne, Illinois from 1924-32. It is well accepted that there is unintended reactivity by participants in randomised controlled trials [2-4]. Placebo control conditions have been developed in pharmacological trials and elsewhere to control for the effects of disappointment at allocation outcome as well as for the placebo effect itself [5,6]. Despite longstanding awareness of reactivity [1], we are aware of only one experimental study measuring the size of the aggregate effect. This study found the effect to be large: approximately 1.5 standard deviations maintained for six months on an objectively ascertained outcome without scope for information bias [7]. However, this dental experiment compared one group, led to believe both that they were in a trial and in receipt of experimental toothpaste, with a second group from whom consent was not obtained and who were unaware of research participation. This design means that the specific effects of trial participation cannot be separated from the broader effects of research participation. There are also many possible explanations for the observed differences including research recruitment, research assessment, randomisation and placebo effects. Of these, assessment reactivity or mere measurement effects have attracted considerable recent attention [8-11] and there is an extensive and advanced literature on placebo effects [6,12,13].

In addition to possible effects in the recruitment phase of a randomised controlled trial (RCT), the process of randomisation itself may be perceived by participants as odd or confusing [2,14]. This uncertainty about how participants view the process of taking part in RCTs leads logically to the question of whether participation in these studies generates sufficient reflection upon behaviour to impact upon the outcomes of interest. An experimental contrast with participation in a non-randomised study is needed to evaluate this possibility.

This study, funded via a project grant from the Australian Research Council, will examine the effects of participants' knowledge of research design, comparing the effects of participation in a cohort study, with participation in a RCT. The behavioural focus of the study will be alcohol consumption. Participant awareness of study design and random allocation will be experimentally manipulated. Two hypotheses will be tested: (1) that knowledge of participation in a randomised study in comparison to a cohort (i.e., a non-experimental) study alone will reduce participants' alcohol consumption; and (2) that knowledge of allocation to an intervention condition in comparison to a control condition in a randomised trial will reduce participants' alcohol consumption. This latter hypothesis investigates whether placebo effects are influenced by expectancies generated as a result of the trial process rather than solely deriving from the perceived properties of the intervention.

## Methods

### Design

**Figure 1:**
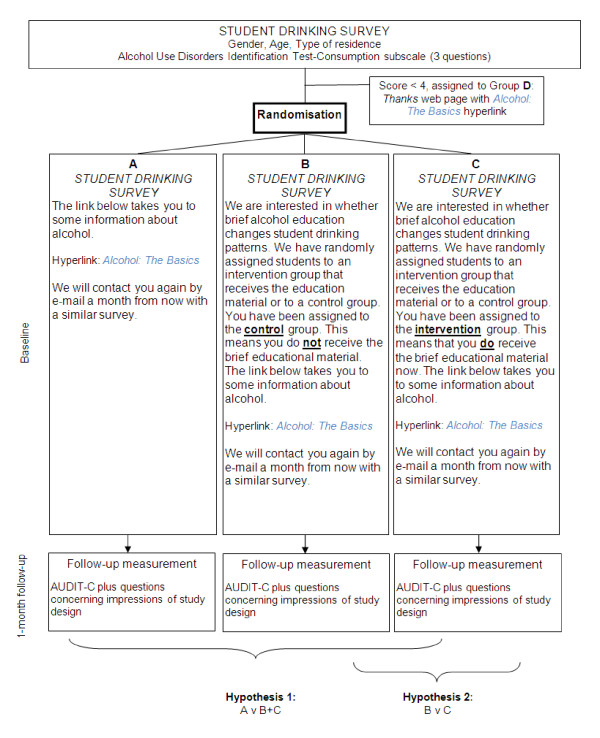
**Study design**.

The study design is a multi-centre three-arm parallel group randomised controlled trial (Figure 1). Ethical approval to conduct the study was granted by the University of Otago Ethics Committee (Protocol number: 10/148).

### Setting

The setting is large public universities in New Zealand and Australia and recruitment and randomisation will be undertaken in waves, one university at a time, with adjustment to sample size estimates after preliminary analyses at each stage. This design is not to be confused with the stepped wedge design, as participants are randomised to the three experimental conditions as they are recruited.

### Procedure

All students will be invited by e-mail to participate in an on-line "Research project on student drinking". Students will be informed that "The study involves the completion of two short web surveys each one month apart" and asked to click on a hyperlink which takes them to the study website (URL) and information form (http://www.behaviourscience.net/InformedConsent.pdf). Clicking on a link to complete the first survey will be taken as consent. A reminder message will be sent two weeks later.

### Screening

**Figure 2:**
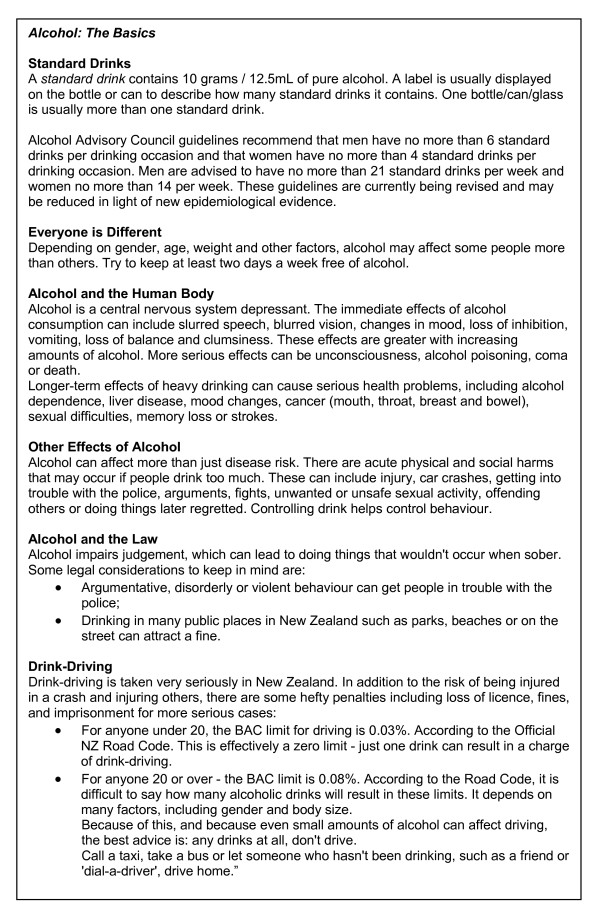
***Alcohol: The Basics *material**.

The baseline survey will be comprised of questions about alcohol intake and other behaviour (available at http://behaviourscience.net/). Any participant whose answers indicate a moderate to high level of alcohol intake (indicated by a score of 4 or more on the AUDIT-C [15]) will be randomised to one of three conditions. Those who score less than 4 on the AUDIT-C will be thanked for participating and provided with a link to *Alcohol: The Basics*, a page containing information about effects of alcohol, safe drinking levels and problems associated with drinking, such as drink-driving (Figure 2).

### Randomisation

Following screening, participants will be randomised without their knowledge to one of three conditions (A-C). Randomisation will be effected by computer using a random number generator. Participants will not be informed that they are participating in a randomised study and given the computerised randomisation, the research team will not know which group each participant is assigned to until after outcomes are assessed. There is thus no opportunity for randomisation to be subverted.

### Interventions

Participants in the three experimental groups will also be presented with the opportunity to access the *Alcohol: The Basics *material via a hyperlink. This material was selected *not *to be effective in promoting behaviour change, and there will be identical levels of encouragement to actually read the alcohol health information in each condition. The differences between groups exist solely in the way the study is described to participants (see Figure 1), namely, in what students are told is the design of the study (cohort or trial), along with their allocation status if randomised to the trial. It should be noted that Figure 1 includes the exact text presented to participants with the differences between conditions B and C emboldened here.

### Outcome measurement

At baseline each participant will be advised they will be sent another survey by email in a month's time. The second survey (which can be viewed at http://behaviourscience.net/) contains eight questions about drinking patterns over the past month and participants' impressions of the study. The primary outcome measures will be the frequency, quantity of alcohol consumption per typical drinking occasion (in standard drinks; 10 g ethanol), volume of alcohol consumed in the preceding four weeks and the incidence of hangover in the same period.

### Sample size estimation

There is no previous research on which to base an expected effect size for this study. Assuming power of 0.80, alpha 0.05, dispersion of 0.2, and a 2-sided test, we would need to analyse 1,946 cases per group at 1 month (i.e., a total N of 5838) we could detect a group difference of 5.5% for comparisons of B v C (Hypothesis 2). This sample size provides additional power to detect an effect of similar size for A v B and C (Hypothesis 1). If the dispersion is greater than anticipated at 0.4, with 5,838 participants at follow-up we will still be able to detect a 7% difference. Accordingly, we will invite all students at the University of Otago (approximately 20,000 individuals), with the expectation of recruiting approximately a third of the total required, which is feasible considering a recent survey at that university [16]. We will then conduct preliminary analyses to estimate an effect size and determine the number to be invited from a second university, and whether a third university is needed.

### Analysis

Groups B and C will be combined for comparison with group A for testing of Hypothesis 1. Groups B and C will be compared to test Hypothesis 2. Alcohol consumption will be analysed using negative binomial regression, with baseline AUDIT-C score as a covariate to account for regression to the mean. Hangover incidence and other dichotomous variables will be analysed with logistic regression. Results will be presented as risk ratios and odds ratios. Participants will be analysed in the group to which they were randomised (intention to treat). Analyses will be stratified by centre to account for the multicentre nature of the trial.

### Attrition

Participants who fail to respond to the e-mail invitation to complete the follow-up questionnaire and the reminder two weeks later will be sent an e-mail inviting them to respond to two questions (frequency and typical occasion quantity) within the body of an e-mail message. These responses will be used to determine whether there are differences at baseline between these individuals and those who completed the follow-up by way of investigating attrition bias.

### Pilot research

A key issue in the design of this experiment is that the alcohol health information should appear credible to all study conditions. This study unavoidably involves the possibility of a placebo effect, in which the belief among the intervention group in the beneficial nature of their allocation itself influences subsequent behaviour. At the same time, it requires the absence of such an effect for the other two groups who receive exactly the same material.

Pilot work, consisting of informant interviews with students has been conducted according to a procedure described in detail elsewhere [17]. We asked participants to complete the baseline survey which included randomisation. They were then provided with hard copies of the survey and asked to write down any comments they had. We asked each participant questions to elicit an affective response including 'What did you think of the questionnaire?' 'How did you feel about answering the questions?' and 'Were there any questions you didn't feel comfortable answering?'. Participants were then asked a series of cognitive questions: 'Were the questions easy to understand?', 'Were the response options reasonable?', 'Can you think of any response options that should be included but weren't?', 'Do you think the option "prefer not to answer" is necessary for any of the questions?', 'Did you notice any wording/spelling mistakes in the questionnaire?'.

### Baseline

Most (14/21) of the pilot participants viewed the baseline survey wholly positively. They said they felt comfortable answering the questions and that the survey was well laid out, straightforward, non-invasive, non-ambiguous and well-designed. Negative comments (4/21) included that it was extremely brief and one participant wondered if we should be asking more questions in order to "get a better understanding" of student drinking. Similarly, another participant did not understand what use the survey would be and suggested we include questions about drinking motives and offences relating to alcohol.

In regard to *Alcohol the Basics*, most participants either did not read or skim read the information. Only four made comments about it: one found it 'especially helpful' in regard to limits; two said there was a 'good level of detail' but one of these suggested adding more information. One participant commented they had 'heard it all before'. There were various comments regarding question 4 ("How often do you have a drink containing alcohol?") being unclear and too general.

There were 10 comments specific to randomisation. Six people found the concept of randomisation 'weird', 'confusing' or 'unusual', however, this was not always negative as one said it was 'unusual but OK'. Two of these comments were in regard to being randomised to the control group but receiving information anyway. The other four were positive about the randomisation process saying they 'felt fine' or 'didn't mind' being randomised.

### Follow-up

There was very little feedback regarding the follow-up survey with over half (11/21) providing no comment. One participant said it was 'extremely short but good'; two thought it connected well to the baseline survey and one said it was similar to the baseline survey. One participant felt uncomfortable answering Question 5 ("What would you say was the design of the study?") but did not explain why. Another person questioned the use of the survey as they did not understand what difference could be shown after four weeks.

All comments and feedback were assessed and the survey was adjusted accordingly. Changes included: adding the demographics questions 1-3 from Baseline (gender, age and living arrangements) to the Follow-up survey; making the drop-down menus larger on screen so respondents did not need to scroll down as far; adding the response option 'don't know' to question 6 of the Follow-Up survey; questions 6 and 7 of the Follow-Up survey were changed from "after completing the survey last month..." to "after completing the previous survey..." as some follow-up surveys would fall in the same month, although four weeks after the baseline survey.

## Discussion

### Ethical considerations

This study aims to determine whether simply taking part in different types of research is enough to differentially decrease participants' consumption of alcohol. While some participants will be under the impression they have received an intervention, in fact all participants will be given access to the same material. The deception is that, in fact, there is no difference between Groups A, B and C except that Group A will be told they are merely completing two surveys, Group B will be told they are in a control group and Group C will be told they are in an intervention group. Upon completion of the study, participants will be sent a hyperlink to a debriefing page describing the study aims and procedures and explaining the need for concealment of the true nature of the study.

### Public health relevance

A key challenge for the health sciences is to develop and evaluate interventions that may have relatively small effects at the individual level, but which, when aggregated across a large population, produce a benefit. The trials required to evaluate such interventions typically involve some of the features to be examined in this trial and the detection of any effects in relation to either hypothesis entails bias. To the extent that they are subject to bias, poor estimates of efficacy and cost-effectiveness will be produced. Although alcohol consumption is chosen as the focal behaviour for the purpose of this study, these experimental manipulations have wider relevance. Accordingly, intervention trials for a range of behaviours, such as physical inactivity, smoking, hazardous drinking, and poor nutrition could benefit from application of the study findings. Similarly, although this study will be undertaken online, it is possible, or indeed likely, that the effects will be greater in magnitude in studies where there is direct contact with researchers [18]. The contribution of study findings therefore extends beyond the development of efforts to minimise bias in online trials. In addition, the research will improve interpretation of existing findings and their use in policy development and clinical practice, which will in turn enhance health and reduce resource misuse.

## Competing interests

The authors declare that they have no competing interests.

## Authors' contributions

JM and KK conceived of the trial and designed it with input from JA, SB and PS. AW and TV conducted the piloting and assisted in the development of the trial materials and procedures under the direction of KK. All have been involved in drafting the manuscript or revising it critically for important intellectual content. All have given final approval of the version to be published.
